# We simply cannot go on being so vague about ‘function’

**DOI:** 10.1186/s13059-018-1600-4

**Published:** 2018-12-18

**Authors:** W. Ford Doolittle

**Affiliations:** Department of Biochemistry and Molecular Biology, Dalhousie University Faculty of Medicine, Halifax, Nova Scotia B3H 4R2 Canada

## Abstract

Function is an onerous concept, as the recent study by Steven Salzberg and colleagues demonstrates. We should be careful and always specific in using the ‘F-word’.

## Introduction

A recent study in *Genome Biology* by the Salzberg laboratory reported on the assembly of a new human gene catalog, based on an exhaustive transcriptomic survey of 31 tissues from hundreds of human subjects [1]. After the removal of transcripts that overlapped with those found in RefSeq or GENCODE databases and additional filtering, they found what appeared to be 224 new protein-coding genes and 116,156 new non-coding transcripts that they deemed to be functional. More surprising is their claim to have also detected over 30 million additional non-functional transcripts, revealing an overwhelming amount of “transcriptional noise” in human cells.

## Functional and non-functional noncoding RNAs

The findings from Salzberg and colleagues could be seen as a decisive blow in the dispute over noncoding RNAs (ncRNAs)—are they functional or are they not? Of course, discretely functional structural RNAs such as ribosomal and transfer RNAs were known even before coding was understood, and there is now an abundance of well-studied small regulatory RNA species. Moreover, at least a few long ncRNAs (lncRNAs) play important developmental and cellular roles, and are as thoroughly documented as many proteins. But most of many genomes is transcribed, albeit infrequently. It is about this process and its products that there is disagreement, and two schools of thought.

The first school, which may be called ‘functionalist’, imagines that these RNAs comprise a vast interconnected network of subtle regulatory and evolutionary capabilities (evolvability), realized and potential. John Mattick and collaborators [2], for instance, consider that we are in the midst of a “conceptual upheaval”, grounded in “the unfolding discovery of previously hidden layers of regulatory RNAs (including many derived from retrotransposon sequences and pseudogenes) and the emerging realization that the genome might not be constructed as a discrete set of protein-coding genes with associated regulatory sequences, but as an interleaved continuum of both coding and *cis*- and *trans*-acting regulatory information.” The second school, which could be called ‘skeptics’, regards ncRNAs (especially lncRNAs) as mostly transcriptional noise. In a recent review, Palazzo and Lee [3] discuss how to determine whether any given ncRNA has a function and advocate that “in the absence of any such data, the appropriate null hypothesis is that the RNA in question is junk.”

The two schools came into conflict in 2012, after investigators associated with the ENCODE project claimed that 80.4% of our genome is functional, and thus we might at last “write the eulogy for junk DNA” [4]. After all, that claim was largely based on evidence that most of our DNA is transcribed, in one tissue or another. Indeed, tissue-specific transcription is considered proof of function in many studies. However, there are several reasons why tissue-specific transcription could happen without providing evidence for tissue-specific ‘function’. Indeed, Graur et al. [5] criticized the ENCODE consortium for often falling into the logical error of “affirming the consequent” (i.e., taking a true statement and invalidly concluding its converse), in particular assuming that because functional genes are transcribed, transcribed regions must be functional genes.

ENCODE investigators responded to critics by admitting that assessments of ‘function’ were not easy to make, and that in the case of low-abundance transcripts it was possible that simple presence is not enough for such ascription. They admitted the need to use multiple biochemical criteria in order to elucidate “genome function in human biology and disease”. Still, the functionalist viewpoint seems at odds with the conclusions of Pertea et al. [1] which, compared to those of Lloyd et al. [6] using machine learning models, are based on very straightforward methods. For instance, unlike Mattick, Salzberg and colleagues dismiss pseudogene transcripts by fiat and declare all protein-noncoding RNAs to be non-functional if they (1) were assembled in fewer than ten samples (of almost 10,000) unless at high levels in these, (2) contained only a single exon, or (3) overlapped known genes (on either strand). By these, and a few additional tests that functionalists might consider arbitrary and biased, they declared that over 30 million transcripts at over 650,000 genomic loci were likely nonfunctional—that is to say, transcriptional noise.

This last concept *is* of course well-grounded. Struhl [7] calculated from first principles that more than 90% of the Pol II initiation events in yeast are noise in the sense of not having a ‘biological function’, by which he presumably meant not honed by natural selection in order to contribute to organismal fitness. Accuracy in any informational transfer process such as transcription comes at a cost and perfect accuracy is unattainable. In any case, the number of ‘mistakes’ surely increases with the number of opportunities to make them, particularly with genome size.

## Addressing the functions of ncRNAs

Statistics and detection methods do matter but the problems are deeper than that and not merely technical, as we might see from the following list of questions we could reasonably ask about any ncRNA and its ‘function’.

1. If a short region localized at the 3′ end of a long ncRNA is under selection to interact with a specific site on another molecule, does that make the whole molecule ‘functional’? What if experiments showed that most of the upstream part could be harmlessly deleted? Would, by similar logic, the presence of one functional gene on a chromosome render the whole chromosome ‘functional’?
2. There are good arguments for something like trypanosomal pan-editing having arisen by ‘constructive neutral evolution’, never being under positive selection and always mildly deleterious to organism fitness, but now ineradicable [8]. Does that make the guide RNAs involved ‘functional’? Some would consider a traits’ function to be that effect for which it originally increased in frequency in an ancestral population, an explanation that would not apply here.
3. Most of mammalian genomes are made up of transposable elements and their decay products. For some elements, transcription is vital for transposition, serving a selfish ‘function’. Is that also a function for us mammals, the element’s ‘hosts’? Are functions ascribable to different levels of selection all to be lumped together? By such logic, we might also declare that genes of viruses making us sick are part of our functional gene repertoire.
4. Sometimes it might be that a stretch of DNA ‘functions’ in spacing and chromosomal structuring: is any RNA accidentally transcribed from it also ‘functional’? Presence of the RNA is *evidence* of an essential function for the DNA and its sequence might even be conserved (because that of the DNA is), but what might this say about the RNA?
5. Kaikkonen and Adelman [9] very recently presented “evidence that the act of transcription and the presence of nascent RNA at a locus is often central to function, rather than specific ncRNA sequences or structures.” If the very act of making RNA contributes to fitness but the RNA made is, again, irrelevant, is the RNA ‘functional’?
6. Presumably RNAs that are lethal—for instance, by serving as a microRNA against an essential gene—have been weeded out by natural selection, so that all ncRNAs are at least not lethally dysfunctional. Is that minimum requirement enough?
7. Mattick and other functionalists would argue that ncRNAs and especially lncRNAs represent evolutionary potential, being co-optable into a host of new regulatory roles. And unquestionably the evolutionary trajectory of complex cells *is* influenced (constrained?) by their content of ncRNAs. Is ‘looking ahead’ like this a function?
8. Humans are all different, phenotypically, and surely some of that is due to differences in the expression levels of different genes during development, some of which is influenced by ncRNAs. But if we do not in consequence have more or fewer children, is this a ‘function’?
9. Any analysis of functionality in human genomes is incomplete if it fails to address the fact that many vertebrate genomes are very much larger, and (as far as we know) also extensively transcribed. How are the facts of comparative genomics to be accommodated?

## Concluding remarks

Deeper than all these questions, but underlying the last in particular, is one about what we mean when we use the ‘F-word’ generally. When we talk of a trait’s ‘function’ do we mean ‘what it does’ or ‘why it is there’? Philosophers have written a lot on this, and the evolutionary biologist John Maynard Smith [10] expressed the difference very well when he wrote “…If we say that the *function* of the heart is to pump blood round the body, we do not mean merely that the heart does, as a matter of fact, pump blood. We mean that the heart evolved *because* it pumped blood; that is, those animals whose hearts were better pumps survived and left more descendants…” (emphasis mine).

Philosophers have also pointed out that ecologists, developmental biologists, physiologists, and (I claim here) molecular biologists and genomicists tend to be satisfied with ‘what it does’ or causal role explanations, whereas evolutionary biologists such as Maynard Smith also require ‘why it’s there’ or selected effect rationales. There may be no absolute right or wrong here, and a good argument could be made for eliminating ‘function’ altogether and replacing it with one of those two concepts, whichever seems appropriate. But it is clearly wrong to use conclusions based on one to ‘refute’ hypotheses based on the other. This is what the publicity around ENCODE did, to the detriment of the credibility of genomic science. So we must be careful to say what we mean if we use the ‘F-word’. We cannot simply complain that such philosophical quibbling muddies the waters. They have never been clear!

## Abbreviations

lncRNA: Long noncoding RNA
ncRNA: Noncoding RNA
